# Clinical Spectrum of Drug-Induced Movement Disorders: A Study of 97 Patients

**DOI:** 10.5334/tohm.587

**Published:** 2020-12-16

**Authors:** Harsh V. Gupta, Rajinder K. Dhamija, Vibhash D. Sharma

**Affiliations:** 1 Department of Neurology, The University of Kansas Health System, Kansas City, Kansas, US; 2 Department of Neurology, Lady Hardinge Medical College, New Delhi, IN

**Keywords:** tremor, tardive, drug-induced, akathisia

To the Editor,

We have read the article from Chouksey *et al* entitled ‘Clinical Spectrum of Drug-Induced Movement Disorders: A Study of 97 Patients’ and would like to felicitate the authors for their work [1].

The authors have published a cross-sectional study of 97 patients with different types of movement disorders associated with drugs. Postural tremor (n = 38) was found to be the second most common movement disorder (MD) associated with drugs. It will be interesting to know whether an isolated postural tremor without kinetic or intention component was disabling to this group of patients. In general, patients with kinetic or intention component find the tremor most disabling which is a common reason for referral to the MD clinic [2]. Patients with drug-induced tremor could also have underlying Essential tremor (ET) which could be difficult to differentiate [3]. As previously suggested, a close follow-up to see whether tremor improves with cessation of the drug could help in differentiating the underlying etiology. Spiral drawing can be a helpful tool because it shows an axis in ET [4]. Another interesting aspect of the study by Chouksey et al is the description of “levetiracetam-induced” postural tremor. To our knowledge, levetiracetam-induced tremor has not been described previously. In a study reporting movement disorders in patients taking anti-epileptic medications, there were no reports of levetiracetam-induced tremor. In that study, valproate and carbamazepine were most associated with tremor, followed by phenytoin [5].

Another rare entity to consider under drug-induced MD is tardive tremor which could be responsive to tetrabenazine. This was reported in 1992 where postural tremor of 3–5 Hz responded to tetrabenazine and did not respond to other drugs such as trihexyphenidyl, propranolol, primidone, benzodiazepines, and levodopa [6]. This tremor got worse with the cessation of tetrabenazine and was associated with other MD such as chorea, dystonia, stereotypy, etc. However, these cases were reported before the FDA approval of DaT scan (dopamine transporter scan) and it is possible that tardive tremor could represent an underlying dystonia leading to tremor because some cases reported improvement with a sensory trick [7].

Finally, a case of acute akathisia following the intramuscular injection of haloperidol has been mentioned [1]. This has an important implication in clinical practice because the acute attack of migraine is often managed with addition of a neuroleptic (prochlorperazine or metoclopramide) which can also lead to acute akathisia in the emergency room (ER) [8]. There is a need to educate the ER physicians and general practitioners about this possible side effect since acute migraine headache is commonly seen in the ER.
